# A high-resolution canopy height model of the Earth

**DOI:** 10.1038/s41559-023-02206-6

**Published:** 2023-09-28

**Authors:** Nico Lang, Walter Jetz, Konrad Schindler, Jan Dirk Wegner

**Affiliations:** 1 EcoVision Lab, Photogrammetry and Remote Sensing, ETH Zürich, Zürich, Switzerland; 2 Department of Computer Science, University of Copenhagen, Copenhagen, Denmark; 3 Department of Ecology and Evolutionary Biology, Yale University, New Haven, CT USA; 4 Institute for Computational Science, University of Zurich, Zürich, Switzerland

**Keywords:** Environmental impact, Ecological modelling, Biogeography, Forest ecology

## Abstract

The worldwide variation in vegetation height is fundamental to the global carbon cycle and central to the functioning of ecosystems and their biodiversity. Geospatially explicit and, ideally, highly resolved information is required to manage terrestrial ecosystems, mitigate climate change and prevent biodiversity loss. Here we present a comprehensive global canopy height map at 10 m ground sampling distance for the year 2020. We have developed a probabilistic deep learning model that fuses sparse height data from the Global Ecosystem Dynamics Investigation (GEDI) space-borne LiDAR mission with dense optical satellite images from Sentinel-2. This model retrieves canopy-top height from Sentinel-2 images anywhere on Earth and quantifies the uncertainty in these estimates. Our approach improves the retrieval of tall canopies with typically high carbon stocks. According to our map, only 5% of the global landmass is covered by trees taller than 30 m. Further, we find that only 34% of these tall canopies are located within protected areas. Thus, the approach can serve ongoing efforts in forest conservation and has the potential to foster advances in climate, carbon and biodiversity modelling.

## Abstract (web summary)

Using a deep learning approach, the authors outline a global canopy height map at 10-m resolution combining publicly available optical satellite images and space-borne LiDAR and show that only 5% of the global landmass is covered by trees taller than 30 m.

## Main

As our society depends on a multitude of terrestrial ecosystem services^1^, the conservation of Earth’s forests has become a priority on the global political agenda^2^. To ensure sustainable development through biodiversity conservation and climate change mitigation, the United Nations have formulated global forest goals that include maintaining and enhancing global carbon stocks and increasing forest cover by 3% between 2017 and 2030^2^. Yet global demand for commodities is driving deforestation, impeding progress towards these ambitious goals^3^. Earth observation and satellite remote sensing play a key role in this context, as they provide the data to monitor the quality of forested area at global scale^4^. However, to measure progress in terms of carbon and biodiversity conservation, novel approaches are needed that go beyond detecting forest cover and can provide consistent information about morphological traits predictive of carbon stock and biodiversity^5^ at global scale. One key vegetation characteristic is canopy height^5,6^.

Mapping canopy height in a consistent fashion at global scale is key to understand *terrestrial ecosystem functions*, which are dominated by vegetation height and vegetation structure^7^. Canopy-top height is an important indicator of biomass and the associated, global *aboveground carbon stock*^8^. At high spatial resolution, canopy height models (CHMs) directly characterize *habitat heterogeneity*^9^, which is why canopy height has been ranked as a high-priority *biodiversity variable* to be observed from space^5^. Furthermore, forests buffer microclimate temperatures under the canopy^10^. While it has been shown that in the tropics, higher canopies provide a stronger dampening effect on microclimate extremes^11^, targeted studies are needed to see if such relationships also hold true at global scale^10^. Thus a homogeneous high-resolution CHM has the potential to advance the modelling of *climate impact* on terrestrial ecosystems and may assist forest management to bolster microclimate buffering as a mitigation service to protect biodiversity under a warmer climate^10^.

Given forests’ central relevance to life on our planet, several new space missions have been developed to measure vegetation structure and biomass. A key mission is the Global Ecosystem Dynamics Investigation (GEDI) operated by NASA (National Aeronautics and Space Administration), which has been collecting full-waveform LiDAR data explicitly for the purpose of measuring vertical forest structure globally, between 51.6° N and S (ref. ^12^). GEDI has unique potential to advance our understanding of the global carbon stock, but its geographical range, and also its spatial and temporal resolutions, are limited. The mission, initially planned to last for two years, collected four years of data from April 2019 to March 2023. The instrument will be stored on the International Space Station and is expected to continue collecting data in fall 2024. Independent of these interruptions, GEDI is a sampling mission expected to cover, at most, 4% of the land surface. By design, the collected samples sparsely cover the surface of the Earth, which restricts the resolution of gridded mission products to 1 km cells^12^. In contrast, satellite missions such as Sentinel-2 or Landsat, which have been designed for a broader range of Earth observation needs, deliver freely accessible archives of optical images that are not as tailored to vegetation structure but offer longer-term global coverage at high spatial and temporal resolution. Sensor fusion between GEDI and multi-spectral optical imagery has the potential to overcome the limitations of each individual data source^13^.

In this work, we describe a deep learning approach to map canopy-top height globally with high resolution, using publicly available optical satellite images as input. We deploy that approach to compute a global canopy-top height product with 10-m ground sampling distance (GSD), based on Sentinel-2 optical images for the year 2020. That global map and the underlying source code and trained models are made publicly available to support conservation efforts and science in disciplines such as climate, carbon and biodiversity modelling. The map can be explored interactively in this browser application: nlang.users.earthengine.app/view/global-canopy-height-2020.

However, estimating forest characteristics such as canopy height or biomass from optical images is a challenging task^14^, as the physical relationships between spectral signatures and vertical forest structure are complex and not well understood^15^. Given the vast amount of data collected by the GEDI mission, we circumvent this lack of mechanistic understanding by harnessing supervised machine learning, in particular end-to-end deep learning. From millions of data examples, our model learns to extract patterns and features from raw satellite images that are predictive of high-resolution vegetation structure. By fusing GEDI observations (that is, RH98, the relative height at which 98% of the energy has been returned) with Sentinel-2 images, our approach enhances the spatial and temporal resolution of the CHM and extends its geographic range to the sub-Arctic and Arctic regions outside of GEDI’s coverage. While retrieval of vegetation parameters with deep learning has been demonstrated regionally and up to country scale^16–19^, we scale it up and process the entire global landmass. This step presents a technical challenge but is crucial to enable operational deployment and ensure consistent, globally homogeneous data.

## Results and discussion

### Deep learning approach

Deep learning is revolutionizing fields ranging from medicine^20^ to weather forecasting^21^ and has great potential to advance environmental monitoring^22,23^, but its application to global remote sensing is technically challenging due to the large data volume^22,24^. Cloud platforms such as Google Earth Engine^25^ simplify the analysis of satellite data but provide a limited set of traditional machine learning tools that depend on manual feature design. To use them, one must sacrifice some flexibility in terms of methods in return for easy access to large data archives and compute power. In particular, canopy height estimation with existing standard tools tends to struggle with the underestimation of tall canopies, as the height estimates saturate around 25 to 30 m (refs. ^26–28^). This is a fairly severe limitation in regions dominated by tall canopies, such as tropical forests, and deteriorates downstream carbon stock estimation, because tall trees have especially high biomass^6^. A further restriction of prior large-scale CHM projects is that they rely on local calibration, which hampers their use in locations without nearby reference data^27,28^. Technically, existing mapping schemes aggregate reflectance data over time but perform pure pixel-to-pixel mapping without regard to local context and image texture.

#### Going global

**Fig. 1:**
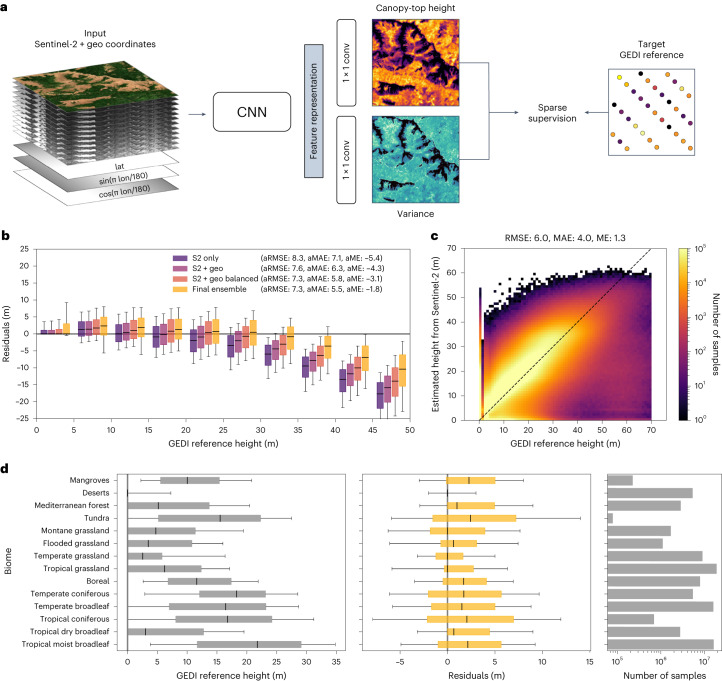
Model overview and global model evaluation on held-out GEDI reference data. **a**, Illustration of the model training process with sparse supervision from GEDI LiDAR. The CNN takes the Sentinel-2 (S2) image and encoded geographical coordinates (lat, lon) as an input to estimate dense canopy-top height and its predictive uncertainty (variance). The two model outputs are estimated from the shared feature representation with separate convolutional layers (conv). **b**, Residual analysis w.r.t. canopy height intervals and ablation study of model components. Negative residuals indicate that estimates are lower than reference values. The boxplot shows the median, the quartiles and the 10th and 90th percentiles (*n* = 88, 332, 537). RMSE, root mean square error; MAE, mean absolute error; ME, mean error (i.e. bias). aRMSE, aMAE and aME are the balanced versions of these metrics, where the metric is computed separately in each 5-m height interval and then averaged across all intervals. **c**, Confusion plot for the final model ensemble, showing good agreement between predictions from Sentinel-2 and GEDI reference. **d**, Biome-level analysis of final ensemble estimates: GEDI reference height, residuals and number of samples per biome. The boxplots show the median, the quartiles and the 10th and 90th percentiles (*n* = 88, 332, 537).

**Extended Data Fig. 1:**
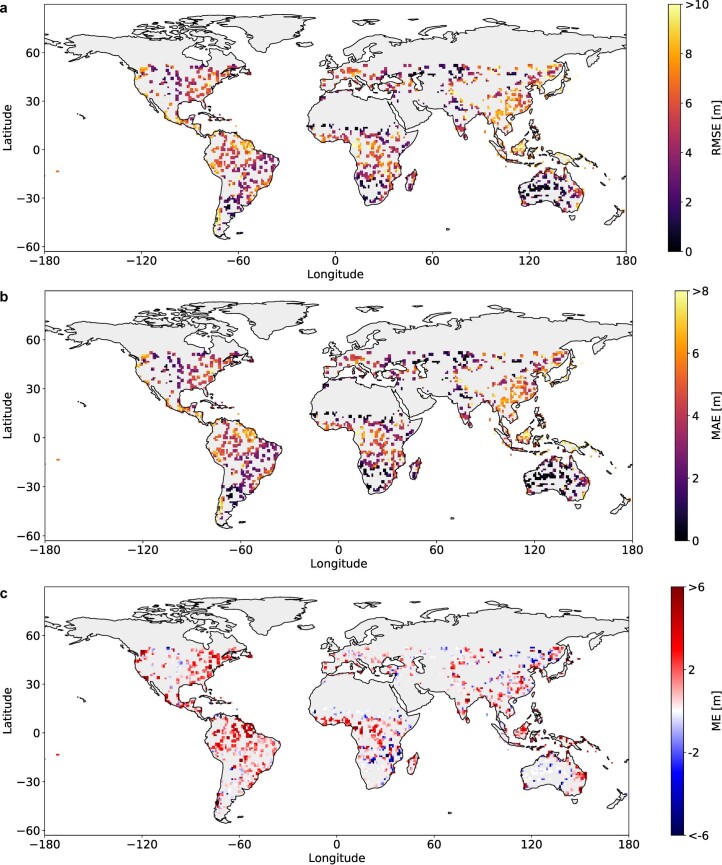
Geographical error analysis on held-out GEDI validation data at 1 degree resolution ( ≈ 111 km on the equator). a) Root mean square error (RMSE). b) Mean absolute error (MAE). c) Mean error (ME), where negative ME means an underestimation bias when the predictions are lower than the reference values.

Here we extend previous regional deep learning methods^16–18^ to a global scale. These methods have been shown to mitigate the saturation of tall canopies by exploiting texture while not depending on temporal features^16^. Previous work has demonstrated that without the ability to learn spatial features, the performance drops substantially especially for the tall canopies^16^. In more detail, our approach employs an ensemble^29^ of deep, (fully) convolutional neural networks (CNN), each of which takes as input a Sentinel-2 optical image and transforms it into a dense canopy height map with the same GSD of 10 m (ref. ^16^) (Fig. 1a). Our unified, global model is trained with sparse supervision, using reference heights at globally distributed GEDI footprints derived from the raw waveforms^30^. A dataset of 600 million samples is constructed by extracting Sentinel-2 image patches of 15 × 15 pixels around every GEDI footprint acquired between April and August in 2019 or 2020. The sparse GEDI data is rasterized to the Sentinel-2 10-m grid by setting the pixel corresponding to the centre of each GEDI footprint to the associated footprint height. In this way, during model training, one can optimize the loss function with respect to (w.r.t.) the model parameters only at valid reference pixels (Fig. 1a); whereas during map generation, the CNN model will nevertheless output a height prediction for every input pixel. To evaluate the model globally, we split the collected dataset at the level of Sentinel-2 tiles. Of the 100 km × 100 km regions defined by the Sentinel-2 tiling, 20% are held out for validation and the remaining 80% are used to train the model (the validation regions and the associated estimation errors are shown in Extended Data Fig. 1).

An important goal of our work is low estimation error for tall vegetation because it indicates potentially high carbon stocks. To that end, we extend the CNN model in three ways (Fig. 1b). First, we equip the model with the ability to learn geographical priors^31^ by feeding it geographical coordinates (in a suitable cyclic encoding) as additional input channels (Fig. 1a). Second, we employ a fine tuning strategy where the sample loss is re-weighted inversely proportional to the sample frequency per 1-m height interval so as to counter the bias in the reference data towards low canopies (which reflects the long-tailed worldwide height distribution, where low vegetation dominates and high values are comparatively rare). Finally, we train an ensemble of CNNs and aggregate estimates from repeated Sentinel-2 observations of the same location, which reduces the underestimation of tall canopies even further. The combination of all three measures yields the best performance. The average root mean square error (aRMSE) of the height estimates, balanced across all 5-m height intervals, is 7.3 m, and the average mean error (aME, that is, bias) is −1.8 m (Fig. 1b). The root mean square error (RMSE) over all validation samples (without height balancing) is 6.0 m, with a bias of 1.3 m (Fig. 1c). The latter is due to a slight overestimation of low canopy heights and is the price we pay for improving the performance on tall canopies (Fig. 1b).

**Extended Data Fig. 2:**
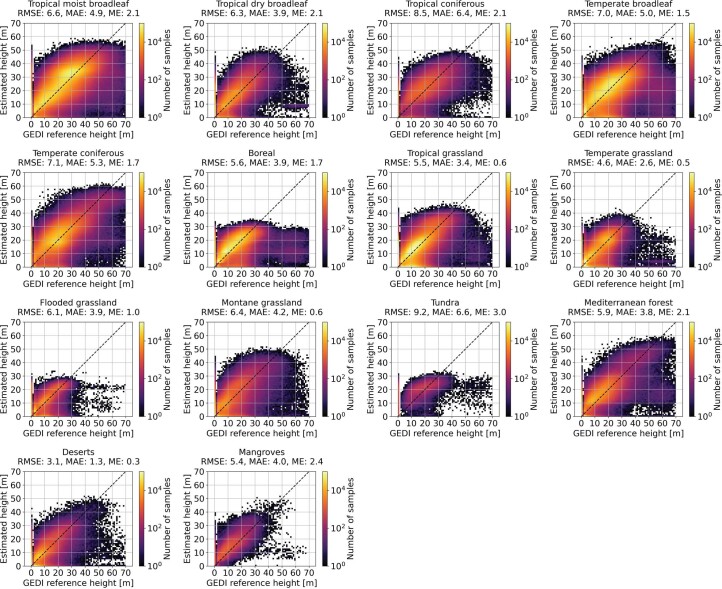
Biome-level evaluation with GEDI reference data. Biome-level confusion plots showing the relationship between GEDI reference data and the estimated canopy top height from Sentinel-2.

A biome-level analysis based on the 14 biomes defined by The Nature Conservancy (www.nature.org) shows how the bias varies across biomes (Fig. 1d and Extended Data Fig. 2). The model is able to correctly identify bare soil in deserts with zero height, with marginal error and no bias. The bias is also low in montane, temperate and tropical grasslands and in Mediterranean and tropical dry broadleaf forest, but higher in flooded grasslands. The most severe overestimation, on average ≈ 2.5 m, is observed for mangroves, tundra and tropical coniferous forests. The highest spread of residuals is observed in tropical and temperate coniferous forests and in the tundra, where we note that the latter is substantially underrepresented in the dataset, as GEDI’s range does not extend beyond 51.6° N. Furthermore, the GEDI reference data in these tundra regions (southern part of Kamchatka Mountain and Forest Tundra and Trans-Baikal Bald Mountain Tundra) appears rather noisy and contaminated with a notable number of outliers (Extended Data Fig. 2).

#### Comparison to existing canopy height estimates

**Extended Data Fig. 3:**
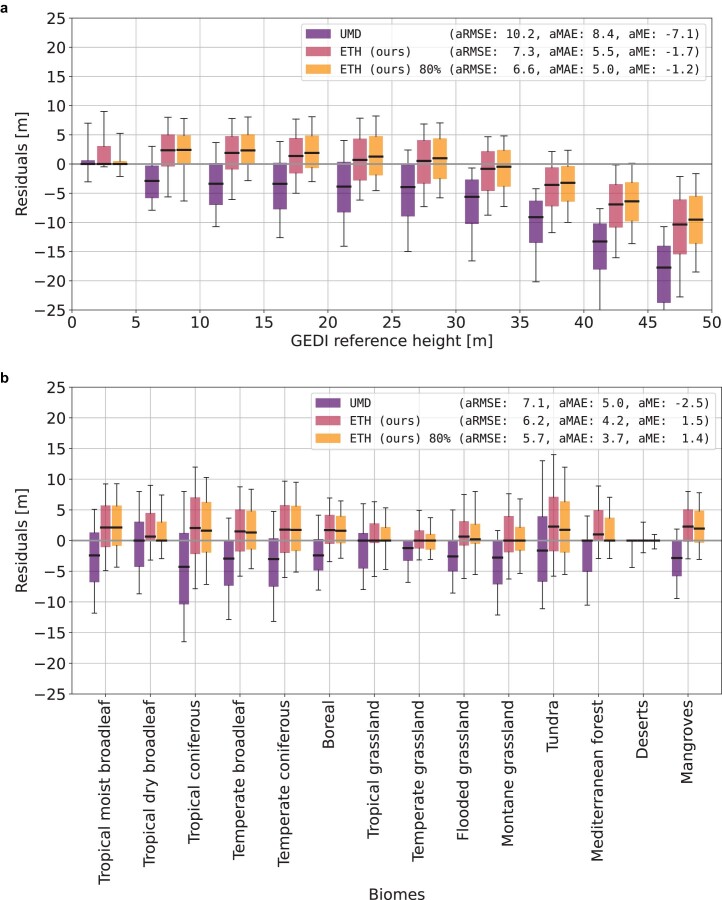
Comparison of canopy top height estimates from ETH (ours) and UMD against the hold-out GEDI validation data. a) Residual analysis w.r.t. GEDI reference canopy height. The boxplot shows the median, the quartiles, and the 10th and 90th percentiles (n=87,406,779 “UMD" and “ETH (ours)"; n=69,925,423 “ETH (ours) 80%"). b) Residual analysis w.r.t. biomes defined by The Nature Conservancy. Negative residuals indicate that estimates are lower than reference values. “ETH (ours) 80%" is a filtered version of our estimates where the 20% of estimates with the highest relative standard deviation are removed. This filtering follows the protocol proposed in previous work using an adaptive threshold depending on the predicted canopy height to preserve the full canopy height range^30^. The distributions over the full height range are compared in Supplementary Information Fig. S2. The boxplot shows the median, the quartiles, and the 10th and 90th percentiles (n=87,406,779 “UMD" and “ETH (ours)"; n=69,925,423 “ETH (ours) 80%").

We compare our estimates with a state-of-the-art global-scale canopy-top height map (henceforth referred to as UMD) that has been derived by combining GEDI data (RH95) with Landsat image composites^27^. This UMD map relies on local model fitting and is created by combining the results of multiple regional models, which means it is not available beyond the GEDI coverage north of 51.6° latitude. For a fair comparison the UMD map with 30 m GSD is re-projected to the same Sentinel-2 10-m grid. Overall, our map reduces the underestimation bias from − 7.1 m to − 1.7 m w.r.t. the hold-out GEDI validation data (in total, 87 million footprints) when averaging the bias across height intervals (Extended Data Fig. 3a). The UMD map underestimates the reference data over the entire height range starting from 5 m canopy height, whereas the underestimation increases for canopy heights >30 m. While the UMD map has negligible bias for low vegetation <5 m, our map tends to overestimate some of the vegetation <5 m. Furthermore, our map has an overestimation bias of ≈ 2 m for heights ranging from 5 to 20 m and a bias of <1 m from 20 to 35 m. Starting from vegetation 35 m tall upwards, the negative bias grows with canopy-top height but is substantially lower compared to the UMD map. The bias also varies across biomes (Extended Data Fig. 3b). In most of the cases where the UMD map tends to underestimate the GEDI reference height, our map tends to overestimate. Exceptions where our map has a low bias of <1 m are: tropical dry broadleaf, tropical grassland, temperate grassland, flooded grasslands, montane grassland and Mediterranean forests. It is worth noting that our global model did not see these validation regions (each 100 × 100 km) during training; in contrast, the UMD approach fits a local model for each region.

#### Evaluation with independent airborne LiDAR

**Extended Data Fig. 4:**
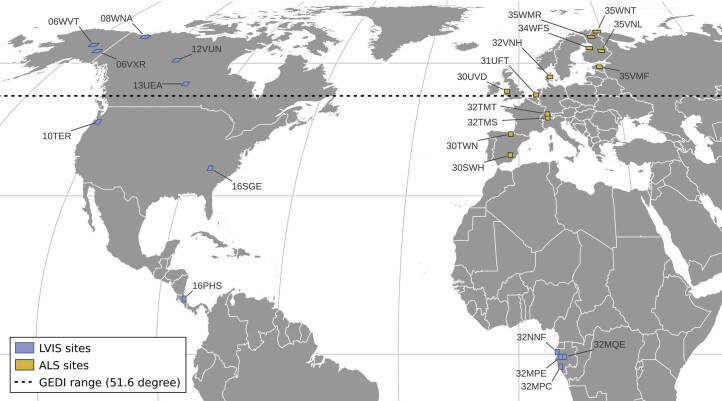
Locations of independent airborne LIDAR campaigns. Independent airborne LIDAR data from 24 regions including 12 regions with LVIS LIDAR and 12 regions with small-footprint airborne laser scanning (ALS) campaigns.

In addition, we compare our final map (and the UMD map) with independent reference data from two airborne LiDAR sources: (1) NASA’s Land, Vegetation, and Ice Sensor (LVIS) airborne LiDAR campaigns^32^, which were designed to deliver canopy-top heights comparable to GEDI^12^ and (2) GEDI-like canopy-top height retrieved from high-resolution canopy height models derived from small-footprint airborne laser scanning (ALS) campaigns in Europe^33^. We report error metrics within 24 Sentinel-2 tile regions (12 each for LVIS and ALS) from 11 countries in North and Central America and Europe (Extended Data Table 1) covering a diverse range of vegetation heights and biomes (Extended Data Fig. 4). In nine out of the 12 regions with UMD map data, our map yields lower random error (RMSE and mean absolute error (MAE)) and bias (mean error). While the UMD map underestimates the airborne LiDAR data in all regions, our map tends to overestimate the reference data in most, but not all, cases. The strongest differences are observed in regions with high average canopy height (that is 28–36 m in the United States (Oregon) and Gabon) where the UMD map has a bias ranging from −19 to −23% w.r.t. the average height, and our map yields a bias of −4% to −13% and 7% to 10%. In the rare cases where the underestimation bias of UMD map is lower than the overestimation bias of ours, we observe qualitatively that our map captures structure within high vegetation, where the UMD map saturates (for example, Netherlands; Extended Data Fig. 7c). Further qualitative comparisons against LVIS and ALS data are presented in Extended Data Fig. 7a–f. Comparing the mean error over regions within the GEDI coverage reveals that our map outperforms the UMD map on all error metrics. The UMD map yields an RMSE of 9.1 m and a bias of −4.5 m (−25.2%) and our map an RMSE of 7.9 m and a bias of 1.7 m (16.2%) (Extended Data Table 2).

Our approach allows us to map beyond the northernmost latitude with GEDI data for which we have 12 regions with independent airborne LiDAR data (Extended Data Table 1). Here we find a mean RMSE of 5.3 m and a bias of 0.5 m (19.4%) (Extended Data Table 2). In the northernmost region in Finland at 70° N latitude, the dominant vegetation structure is captured in our map with an RMSE of 3.0 m and a bias of 0.5 m (17.1%) (for example, Extended Data Fig. 8a). In other words, our estimates agree well with independent LVIS and ALS data, even outside the geographic range of the GEDI training data (qualitative examples in Extended Data Fig. 8a–f).

### Modelling predictive uncertainty

Whereas deep learning models often exhibit high predictive skill and produce estimates with low overall error, the uncertainty of those estimates is typically not known or unrealistically low (that is, the model is over-confident)^34^. But reliable uncertainty quantification is crucial to inform downstream investigations and decisions based on the map content^8^; for example, it can indicate which estimates are too uncertain and should be disregarded^30^. To afford users of our CHM a trustworthy, spatially explicit estimate of the map’s uncertainty, we integrate probabilistic deep learning techniques. These methods are specifically designed to quantify also the predictive uncertainty, taking into account, on the one hand, the inevitable noise in the input data and, on the other hand, the uncertainty of the model parameters resulting from the lack of reference data for certain conditions^35^. In particular, we independently train five deep CNNs that have identical structure but are initialized with different random weights. The spread of the predictions made by such a model ensemble^29^ for the same pixel is an effective way to estimate model uncertainty (also known as epistemic uncertainty), even with small ensemble size^36^. Each individual CNN is trained by maximizing the Gaussian likelihood rather than minimizing the more widely used squared error. Consequently, each CNN outputs not only a point estimate per pixel but also an associated variance that quantifies the uncertainty of that estimate (a.k.a. its aleatoric uncertainty)^35^.

During inference, we process images from ten different dates (satellite overpasses) within a year at every location to obtain full coverage and exploit redundancy for pixels with multiple cloud-free observations. Each image is processed with a randomly selected CNN within the ensemble, which reduces computational overhead and can be interpreted as natural test-time augmentation, known to improve the calibration of uncertainty estimates with deep ensembles^37^.

**Extended Data Fig. 5:**
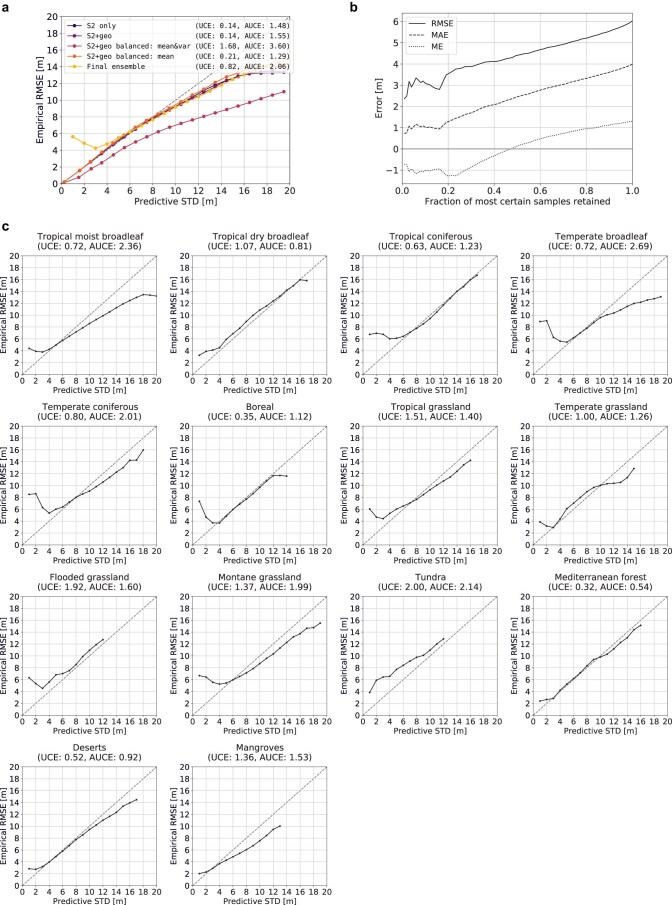
Evaluation of the estimated uncertainty. a) Calibration plot showing the relationship between the estimated predictive uncertainty and the empirical error. b) Improvement of overall error metrics when filtering out the most uncertain canopy height predictions with the help of the estimated predictive uncertainty. c) Biome-level calibration plots, showing the relationship between the estimated predictive uncertainty and the empirical error w.r.t. GEDI reference data.

Finally, we use the estimated aleatoric uncertainties to merge redundant predictions from different imaging dates by weighted averaging proportional to the inverse variance. While inverse-variance weighting is known to yield the lowest expected error^38^, we observe a deterioration of the uncertainty calibration for low values (<4 m standard deviation in Extended Data Fig. 5a). We also note that uncertainty calibration varies per biome (Extended Data Fig. 5c), so it may be advisable to re-calibrate in post-processing depending on the intended application and region of interest. Despite these observations, the estimated predictive uncertainty correlates well with the empirical estimation error and can therefore be used to filter out inaccurate predictions, thus lowering the overall error at the cost of reduced completeness (Extended Data Fig. 5b). For example, by filtering out the 20% most uncertain canopy height estimates, overall RMSE is reduced by 13% (from 6.0 m to 5.2 m) and the bias is reduced by 23% (from 1.3 m to 1.0 m).

Interestingly, the tendency to overestimate some of the low vegetation <5 m can also be removed by using our estimated uncertainty to filter out, for example, the 20% of validation points with the highest relative standard deviation (‘ETH (ours) 80%’ in Extended Data Fig. 3a). Here we follow the filtering protocol proposed in previous work using an adaptive threshold depending on the predicted canopy height to preserve the full canopy height range^30^. The ability to identify erroneous estimates based on the predictive uncertainty is a unique characteristic of our proposed methodology and allows us to reduce random errors and biases (Extended Data Fig. 3).

### Global canopy height map

The model has been deployed globally on the Sentinel-2 image archive for the year 2020 to produce a global map of canopy-top height. To cover the global landmass ( ≈ 1.3 × 10^12^ pixels at the GSD of Sentinel-2), a total of ≈ 160 terabytes of Sentinel-2 image data are selected for processing. This required ≈ 27,000 graphics processing unit (GPU) hours ( ≈ 3 GPU years) of computation time, parallelized on a high-performance cluster to obtain the global map in ten days real time.

**Fig. 2:**
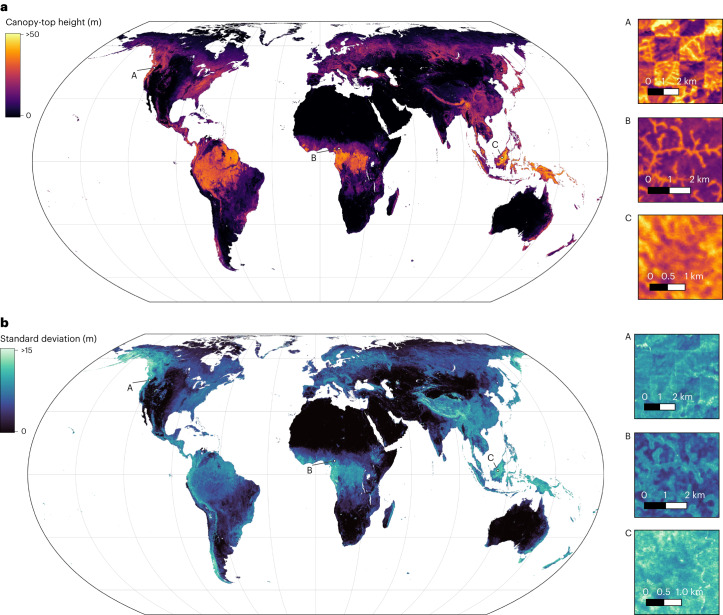
Global canopy height map for the year 2020. The underlying data product, estimated from Sentinel-2 imagery, has 10-m ground sampling distance and is visualized in Equal Earth projection. **a**, Canopy-top height. **b**, Predictive standard deviation of canopy-top height estimates. Location A details harvesting patterns in Washington state, United States. Location B shows gallery forests along permanent rivers and ground water in the forest–savannah of northern Cameroon. Location C shows tropical broadleaf forest in Borneo, Malaysia.

The new dense canopy height product at 10-m GSD makes it possible to gain insights into the fine-grained distribution of canopy heights anywhere on Earth and the associated uncertainty (Fig. 2). Three example locations (A–C in Fig. 2) demonstrate the level of canopy detail that the map reveals, ranging from harvesting patterns from forestry in Washington state, United States (A), through gallery forests along permanent rivers and ground water in the forest–savannah of northern Cameroon (B), to dense tropical broadleaf forest in Borneo, Malaysia (C).

Also at large scale, the predictive uncertainty is positively correlated with the estimated canopy height (Fig. 2b). Still, some regions such as Alaska, Yukon (northwestern Canada) and Tibet exhibit high predictive uncertainty, which cannot be explained only by the canopy height itself. The two former lie outside of the GEDI coverage, so the higher uncertainty is probably due to local characteristics that the model has not encountered during training. The latter indicates that also within the GEDI range, there are environments that are more challenging for the model, for example, due to globally rare ecosystem characteristics not encountered elsewhere. Ultimately, all three regions might be affected by frequent cloud cover (and snow cover), limiting the number of repeated observations. Qualitative examples with high uncertainty, but reasonable canopy-top height estimates, for Alaska are presented in Extended Data Fig. 8e,f.

**Fig. 3:**
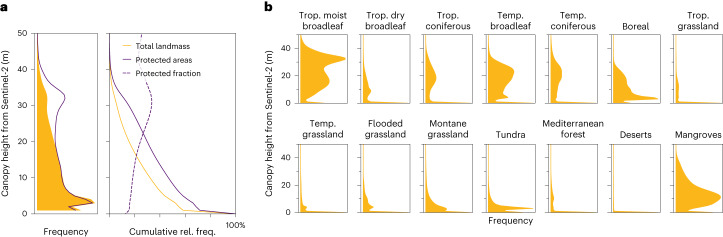
Global canopy height distributions of the entire landmass, protected areas and biomes. **a**, Frequency distribution and cumulative distribution (relative frequency) for the entire global landmass and within protected areas (according to WDPA^39^) and fraction of vegetation above a certain height that is protected. **b**, Biome-level frequency distribution of canopy heights according to 14 terrestrial ecosystems defined by The Nature Conservancy. Urban areas and croplands (based on ESA WorldCover^58^) have been excluded. Abbreviations are used for tropical (trop.) and temperate (temp.) biomes. Supplementary Fig. 1 shows the distributions for canopy heights >1 m.

**Extended Data Fig. 6:**
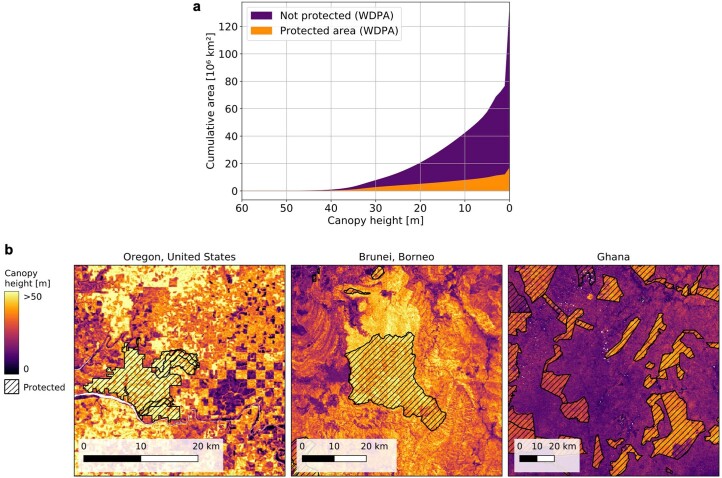
Protected area analysis according to the World Database on Protected Areas (WDPA)^39^. a) Cumulative area covered by vegetation above a given height in protected areas and unprotected areas. The sum at height 0 equals the area of the global landmass (excluding Antarctica). b) Examples where the dense canopy height map reveals the spatial patterns of protected areas. Left: “Devil’s Staircase Wilderness" containing federally protected old-growth forest stands in the Oregon Coast Range. Center: “Ulu Temburong National Park" in Brunei, Borneo, established in 1991. Right: Protected areas in Ghana that indicate the strong impact of protection measures on the growing vegetation.

**Extended Data Fig. 7:**
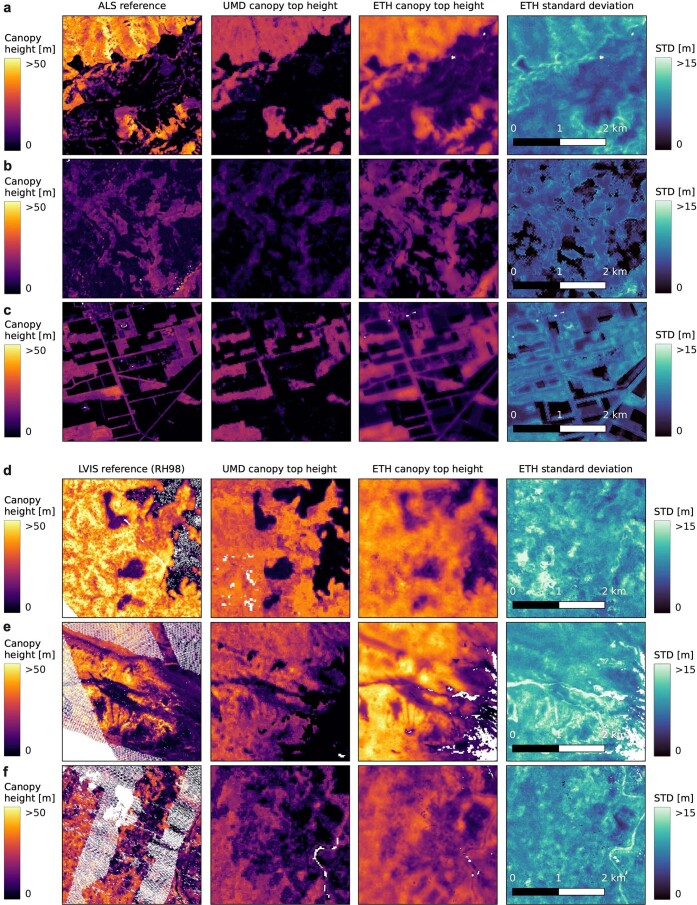
Qualitative examples comparing UMD and ETH (ours) against GEDI-like canopy top height derived from small-footprint airborne laser scanning (ALS) campaigns (a-c) and LVIS LIDAR (d-f). a) Switzerland (from tile region 32TMT with average height 14.1m). Our map with RMSE: 8.0m, bias: 1.5 m (10.5%) outperforms the UMD map with RMSE: 10.1m, bias: -5.4 m (-37.4%). b) Spain (from tile region 30SWH with low average height 6.0m). Our map with RMSE: 4.7m, bias: 0.5 m (7.7%) outperforms the UMD map with RMSE: 5.2m, bias: -3.3 m (-54.9%). c) Netherlands (from tile region 31UFT with average height 6.6m). Our map with RMSE: 6.4m, bias: 3.9 m (59.9%) yields higher error than the UMD map with RMSE: 5.9m, bias: -2.2 m (-37.5%). In both b) and c), the UMD map underestimates high vegetation and misses small structures in low vegetation areas. d) Gabon, Lopé National Park (from the tile region 32MQE with the highest average height of 36.2 m). Our map with RMSE: 8.9m, bias: -4.8 m (-13.3%) outperforms the UMD map with RMSE: 11.4m, bias: -7.9 m (-21.8%). e) US, Oregon (from tile region 10TER with an average height of 28.6m). Our map with RMSE: 9.6m, bias: 2.9m, (10.0%) outperforms the UMD map with RMSE: 12.0 m, bias: -5.4 m (-19.3%). f) Costa Rica (from tile region 16PHS with average height 16.7m). Our map with RMSE: 9.2m, bias: 5.9 m (35.4%) yields a higher error than the UMD map with RMSE: 8.1m, bias: -3.0 m (-18.0%).

**Extended Data Fig. 8:**
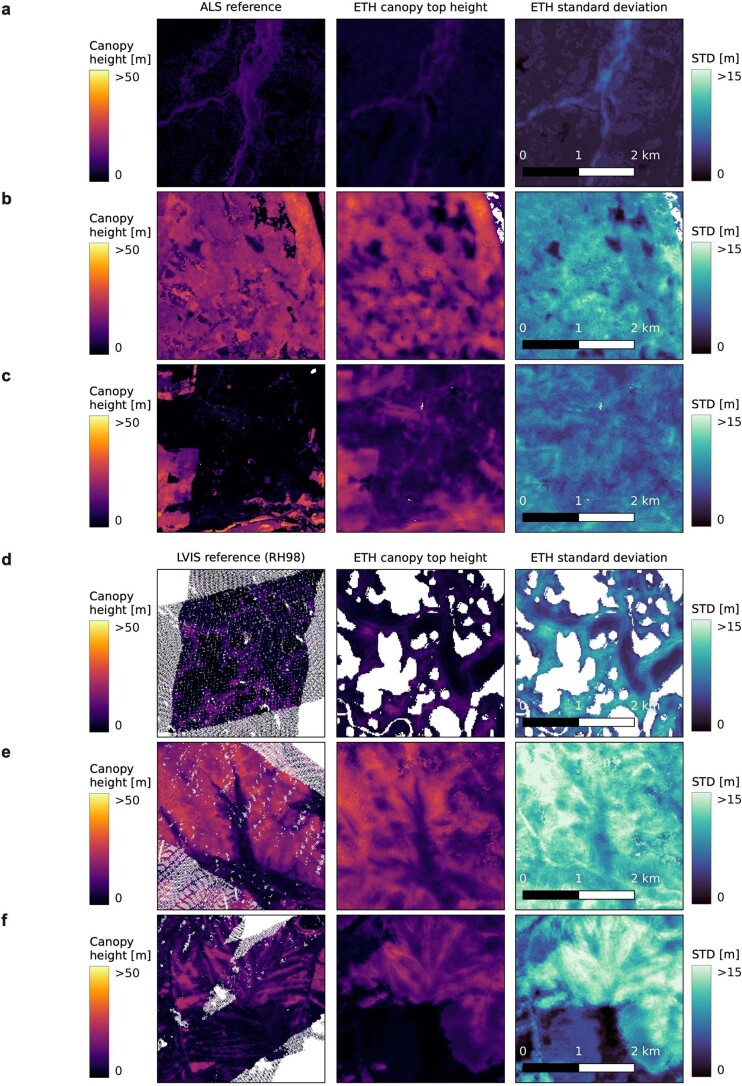
Qualitative examples beyond GEDI coverage (that is north of 51.6^∘^ latitude) against GEDI-like canopy top height derived from small-footprint airborne laser scanning (ALS) campaigns (a-c) and LVIS LIDAR (d-f). a) Finland (from tile region 35WNT with average height 3.0m). Our map yields an RMSE: 3.0 m and bias: 0.5 m (17.1%). b) Finland (from tile region 35VNL with average height 15.3m). Our map yields an RMSE: 5.7 m and bias: -2.6 m (-17.2%). c) Wales (from tile region 30UVD with average height 3.3m). An error case with an RMSE: 7.3 m and bias: 5.7 m (171.1%). Our map overestimates the ALS reference data, especially in low vegetation areas. d) Canada (from tile region 08WNA with average height 4.3m). Our map yields an RMSE: 2.8 m and bias: 0.7 m (16.4%). e) US, Alaska (from tile region 06VXR with average height 9.3m). Our map yields an RMSE: 6.4 m and bias: 2.7 m (29.2%). f) US, Alaska (from tile region 06WVT with average height 6.8m). Our map yields an RMSE: 5.3 m and bias: 0.6 m (8.7%). Both e) and f) show high predictive uncertainty, yet the canopy top height estimates are reasonable.

Our new dataset enables a full, worldwide assessment of coverage of the global landmass with vegetation. Doing this for a range of thresholds recovers an estimate of the global canopy height distribution (for the year 2020; Fig. 3a and Extended Data Fig. 6a). We find that an area of 53.6 × 10^6^ km^2^ (41% of the global landmass) is covered by vegetation with >5 m canopy height, 39.6 × 10^6^ km^2^ (30%) by vegetation >10 m and 6.7 × 10^6^ km^2^ (5%) by vegetation >30 m (Fig. 3b).

We see that protected areas (according to the World Database on Protected Areas, WDPA^39^) tend to contain higher vegetation compared to the global average (Fig. 3a). Furthermore, 34% of all canopies >30 m fall into protected areas (Fig. 3a). Extended Data Fig. 6b provides examples of protected areas that show good agreement with mapped canopy height patterns. This analysis highlights the relevance of the new dataset for ecological and conservation purposes. For instance, canopy height and its spatial homogeneity can serve as an ecological indicator to identify forest areas with high integrity and conservation value. That task requires both dense area coverage at reasonable resolution and a high saturation level to locate very tall vegetation.

Finally, our new map makes it possible to analyse the exhaustive distributions of canopy heights at the biome level, revealing characteristic frequency distributions and trends within different types of terrestrial ecosystem (Fig. 3b). While, for instance, the canopy heights of tropical moist broadleaf forests follow a bimodal distribution with a mode >30 m, mangroves have a unimodal distribution with a large spread and heights ranging up to >40 m. Notably, our model has learned to predict reasonable canopy heights in tundra regions, despite scarce and noisy reference data for that ecosystem.

### Discussion

The evaluation with hold-out GEDI reference data and the comparison with independent airborne LiDAR data show that the presented approach yields a new, carefully quality-assessed state-of-the-art global map product that includes quantitative uncertainty estimates. But the retrieval of canopy height from optical satellite images remains a challenging task and has its limitations.

#### Limitations and map quality

We note that despite the nominal 10-m ground sampling distance of our global map, the effective spatial resolution at which individual vegetation features can be identified is lower. As a consequence of the GEDI reference data used to train the model, each map pixel effectively indicates the largest canopy-top height within a GEDI footprint ( ≈ 25 m diameter) centred at the pixel. Two subtle reasons further impact the effective resolution, compared to a map learned from dense, local reference data (for example, airborne LiDAR^16^): the sparse supervision means that the model never explicitly sees high-frequency variations of the canopy height between adjacent pixels, and misalignments caused by the geolocation uncertainty (15–20 m) of the GEDI version 1 data^12,40,41^ introduce further noise. While at present, we do not see this as severely limiting the utility of the map, in the future one could consider extending the method with techniques for guided super resolution^42^ to better preserve small features visible in the raw Sentinel-2 images, such as canopy gaps. Furthermore, using the latest GEDI release version with improved geolocation accuracy^41^ may improve the measured performance.

Regarding map quality, besides minor artefacts in regions with persistent cloud cover, we observe tiling artifacts at high latitudes in the northern hemisphere. The systematic inconsistencies at tile borders point at degradation of the absolute height estimates, possibly caused by a lack of training data for particular, locally unique vegetation structures. Interestingly, it appears that a notable part of these errors are constant offsets between the tiles.

Whereas our approach substantially reduces the error for tall canopies representing potentially high carbon stocks, we observe a tendency to overestimate some areas with very low canopy heights (<5 m). However, future downstream applications may use additional land cover masks or predictive uncertainty to identify and filter these biased estimates. The modelled spatially explicit uncertainty can be used to identify erroneous estimates. On a global scale, we observe that some regions are subject to overall high predictive uncertainty including tropical regions such as Papua New Guinea, but also regions in northern latitudes, for example, Alaska. This shows the importance of modelling the predictive uncertainty to allow transparent and informed use of the canopy height map. It also indicates that there are limits when using optical images as the only predictor for canopy height estimation and that future work could explore the combination with additional predictive environmental data to resolve ambiguities in visual observations.

#### Potential applications

**Fig. 4:**
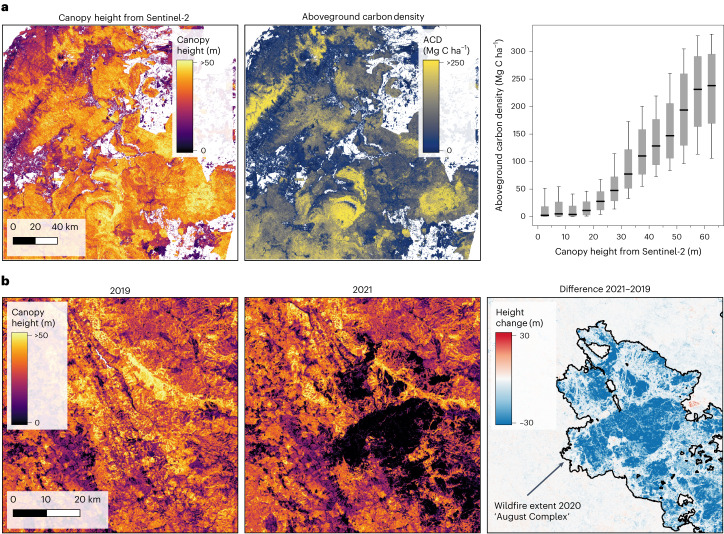
Examples for potential applications. **a**, Biomass and carbon stock mapping. In Sabah, northern Borneo, canopy height estimated from Sentinel-2 optical images correlates strongly with aboveground carbon density (ACD) from a targeted airborne LiDAR campaign^47^ (5.3812° N, 117.0748° E). The boxplot shows the median, the quartiles and the 10th and 90th percentiles (*n* = 339, 835, 325). **b**, Monitoring environmental damages. In 2020, wildfires destroyed large areas of forests in northern California. The difference between annual canopy height maps is in good agreement with the wildfire extent mapped by the California Department of Forestry and Fire Protection (40.1342° N, 123.5201° W).

In the context of the GEDI mission goals^12^, our presented canopy height map may be used to fill the gaps in the gridded products at 1-km resolution where no GEDI tracks are available^43,44^. According to the Global Forest Resources Assessment 2020^45^, 31% of the global land area is covered by forests. Whereas our new global canopy height map can contribute to global forest cover estimates, such a forest definition relies not solely on canopy height, but also on connectivity and includes areas with trees likely to reach a certain height. Therefore, these derivations require more in situ data and threshold calibration. Furthermore, there are at least two major downstream applications that the new high-resolution canopy height dataset can help to advance at global scale, namely biomass and biodiversity modelling. Furthermore, our model can support monitoring of forest disturbances. Canopy-top height is a key indicator to study the global aboveground carbon stock stored in the form of biomass^8^. On a local scale, we compare our canopy height map with dense aboveground carbon density data^46^ that was produced by a targeted airborne LiDAR campaign in Sabah, northern Borneo^47^ (Fig. 4a). We observe that for natural tropical forests, the spatial patterns agree well and that our canopy height estimates from Sentinel-2 are predictive of carbon density even in tropical regions, with canopy heights up to 65 m. Notably, the relationship between carbon and canopy height is sensitive up to ≈ 60 m. We note that although it is technically possible to map biomass at 10-m ground sampling distance, this may not be meaningful in regions such as the tropics, with dense vegetation where single tree crowns may exceed a 10-m pixel. It is rather recommended to model biomass at coarser spatial resolutions (for example 0.25 ha) suitable to capture the variation of dense vegetation areas. Nevertheless, high-resolution canopy height data has great potential to improve global biomass estimates by providing descriptive statistics of the vegetation structure within local neighbourhoods.

We further demonstrate that our model can be deployed annually to map canopy height change over time, for example, to derive changes in carbon stock and estimate carbon emissions caused by global land-use changes, at present mainly deforestation^48^. Annual canopy height maps are computed for a region in northern California where wildfires have destroyed large areas in 2020 (Fig. 4b). Our automated change map corresponds well with the mapped fire extent from the California Department of Forestry and Fire Protection (www.fire.ca.gov), while at the same time the annual maps are consistent in areas not affected by the fires, where no changes are expected. While the sensitivity of detectable changes such as annual growth might be limited by the model accuracy and remains to be evaluated (for example, with repeated GEDI shots or LiDAR campaigns), such high-resolution change data may potentially help to reduce the high uncertainty of emissions estimates that are reported in the annual Global Carbon Budget^48^. It is worth mentioning that the presented approach yields reliable estimates based on single cloud-free Sentinel-2 images. Thus, its potential for monitoring changes in canopy height is not limited to annual maps but to the availability of cloud-free images that are taken at least every five days globally. This high update frequency makes it relevant for, for example, real-time deforestation alert systems, even in regions with frequent cloud cover.

A second line of potential applications includes biodiversity modelling as the high spatial resolution of the canopy height model brings about the possibility to characterize habitat structure and vegetation heterogeneity, known to promote a number of ecosystem services^49^ and to be predictive of biodiversity^50,51^. The relationship between heterogeneity and species diversity is founded in niche theory^50,51^, which suggests that heterogeneous areas provide more ecological niches for different species to co-exist. Our dense map makes it possible to study second-order homogeneity^9^ (which is not easily possible with sparse data like GEDI) and down to a length scale of 10 to 20 m.

Technically, our dense high-resolution map makes it a lot easier for scientists to intersect sparse sample data, for example, field plots, with canopy height. To make full use of scarce field data in biomass or biodiversity research, dense complementary maps are a lot more useful: when pairing sparse field samples with other sparsely sampled data, the chances of finding enough overlap are exceedingly low; whereas pairing them with low-resolution maps risks biases due to the large-scale difference and associated spatial averaging.

## Conclusion

We have developed a deep learning method for canopy height retrieval from Sentinel-2 optical images. That method has made it possible to produce a globally consistent, dense canopy-top height map of the entire Earth, with 10-m ground sampling distance. Besides Sentinel-2, the GEDI LiDAR mission also plays a key role as the source of sparse but uniquely well-distributed reference data at global scale. Compared to previous work that maps canopy height at global scales^27^, our model substantially reduces the overall underestimation bias for tall canopies. Our model does not require local calibration and can therefore be deployed anywhere on Earth, including regions outside the GEDI coverage. Moreover, it also delivers spatially explicit estimates for the predictive uncertainties of the retrieved canopy heights. As our method, once trained, needs only image data, maps can be updated annually, opening up the possibility to track the progress of commitments made under the United Nation’s global forest goals to enhance carbon stock and forest cover by 2030^2^. At the same time, the longer the GEDI mission will collect on-orbit data, the denser the reference data for our approach will become, which can be expected to diminish the predictive uncertainty and improve the effective resolution of its estimates.

As a possible future extension, our model could be extended to map other vegetation characteristics^17^ at global scale. In particular, it appears feasible to densely map biomass by retraining with GEDI L4A biomass data^8^ or by adding additional data from planned future space missions^52^.

Whereas deep learning technology for remote sensing is continuously being refined by focusing on improved performance at regional scale, its operational utility has been limited by the fact that it often could not be scaled up to global coverage. Our work demonstrates that if one has a way of collecting globally well-distributed reference data, modern deep learning can be scaled up and employed for global vegetation analysis from satellite images. We hope that our example may serve as a foundation on which new, scalable approaches can be built that harness the potential of deep learning for global environmental monitoring and that it inspires machine learning researchers to contribute to environmental and conservation challenges.

## Methods

### Data

This work builds on data from two ongoing space missions: the Copernicus Sentinel-2 mission operated by the European Space Agency (ESA) and NASA’s Global Ecosystem Dynamics Investigation (GEDI). The Sentinel-2 multi-spectral sensor delivers optical images covering the global landmass with a revisit time of, at most, five days on the equator. We use the atmospherically corrected L2A product, consisting of 12 bands ranging from the visible and near infrared to the short wave infrared. While the visible and near infrared bands have 10-m GSD, the other bands have a 20-m or 60-m GSD. For our purposes, all bands are upsampled with cubic interpolation to obtain a data cube with 10-m ground sampling distance. The GEDI mission is a space-based full-waveform LiDAR mounted on the International Space Station and measures vertical forest structure at sample locations with a 25-m footprint, distributed between 51.6° N and S. We use footprint-level canopy-top height data derived from these waveforms as sparse reference data^30,53^. The canopy-top height is defined as RH98, the relative height at which 98% of the energy has been returned, and was derived from GEDI L1B version 1 waveforms^54^ collected between April and August in the years 2019 and 2020.

To train the deep learning model, a global training dataset has been constructed within the GEDI range by combining the GEDI data and the Sentinel-2 imagery. For every Sentinel-2 tile, we select the image with the least cloud coverage between May and September 2020. Thus, the model is trained to be invariant against phenological changes within this period but is not designed to be robust outside of this period for regions experiencing high seasonality. Ultimately, the annual maps are computed on Sentinel-2 images from the same period. Image patches of 15 × 15 pixels (that is, 150 m × 150 m on the ground) are extracted from these images at every GEDI footprint location. Therefore, the GEDI data are rastered to the Sentinel-2 pixel grid by setting the canopy height reference value of the pixel that corresponds to the centre of the GEDI footprint. Locations for which the image patch is cloudy or snow covered are filtered out from the dataset. To correct noise injected by the geolocation uncertainty of the GEDI version 1 data^41^, we use the Sentinel-2 L2A scene classification and assign 0 m canopy height to footprints located in the categories ‘not vegetated’ or ‘water’. This procedure also addresses the slight positive bias due to slope in the GEDI reference data^30^. Overall, the resulting dataset contains 600 × 10^6^ samples globally distributed within the GEDI range. All samples located within 20% of the Sentinel-2 tiles in that range (each 100 × 100 km) are set aside as validation data (Extended Data Fig. 1).

A second evaluation is carried out w.r.t. canopy-top heights independently derived from airborne LiDAR data from two sources. This includes NASA’s LVIS. LVIS is a large-footprint full-waveform LiDAR from which the LVIS L2 height metric RH98 is rastered to the Sentinel-2 10-m grid. The second source is high-resolution canopy height models (1-m GSD) derived from small-footprint ALS campaigns^33^. To derive a comparable ‘GEDI-like’ canopy-top height metric within the 25-m footprint, we first run a circular max-pooling filter with a 12-m radius at the 1-m GSD resolution with a stride of 1 pixel (that is 1 m) before we resample the canopy height models to the Sentinel-2 10-m GSD (Supplementary Fig. 5 illustration). This processing is necessary as the maximum canopy height depends on the footprint size and avoids the comparison of systematically different canopy height metrics. Locations of the LVIS and ALS data are visualized in Extended Data Fig. 4.

### Deep fully convolutional neural network

Our model is based on the fully convolutional neural network architecture proposed in prior work^16^. The architecture employs a series of residual blocks with separable convolutions^55^ without any downsampling within the network. The sequence of learnable 3 × 3 convolutional filters is able to extract not only spectral but also textural features. To speed up the model for deployment at global scale, we reduce its size, setting the number of blocks to eight and the number of filters per block to 256. This speeds up the forward pass by a factor of ≈ 17 compared to the original, larger model. In our tests, the smaller version did not cause higher errors in an early phase of training. When trained long enough, a larger model with higher capacity may be able to reach lower prediction errors, but the higher computational cost of inference would limit its utility for repeated, operational use. The model takes the 12 bands from Sentinel-2 L2A product and the cyclic encoded geographical coordinates per pixel as input for a total of 15 input channels. Its outputs are two channels with the same spatial dimension as the input, one for the mean height and one for its variance (Fig. 1). Because the architecture is fully convolutional, it can process arbitrarily sized input image patches, which is useful when deploying at large scale.

### Model training with sparse supervision

Formally, canopy height retrieval is a pixel-wise regression task. We train the regression model end to end in supervised fashion, which means that the model learns to transform raw image data into spectral and textural features predictive of canopy height, and there is no need to manually design feature extractors (Supplementary Fig. 3). We train the convolutional neural network with sparse supervision, that is, by selectively minimizing the loss (equation (1)) only at pixel locations for which there is a GEDI reference value. Before feeding the 15-channel data cube to the CNN, each channel is normalized to be standard normal, using the channel statistics from the training set. The reference canopy heights are normalized in the same way, a common pre-processing step to improve the numerical stability of the training. Each neural network is trained for 5,000,000 iterations with a batch size of 64, using the Adam optimizer^56^. The base learning rate is initially set to 0.0001 and then reduced by factor 0.1 after 2,000,000 iterations and again after 3,500,000 iterations. This schedule was found to stabilize the uncertainty estimation.

### Modelling the predictive uncertainty

Modelling uncertainty in deep neural networks is challenging due to their strong nonlinearity but crucial to build trustworthy models. The approach followed in this work accounts for two sources of uncertainty, namely the data (aleatoric) and the model (epistemic) uncertainty^35^. The uncertainty in the data, resulting from noise in the input and reference data, is modelled by minimizing the Gaussian negative log likelihood (equation (1)) as a loss function^35^. This corresponds to independently representing the model output at every pixel *i* as a conditional Gaussian probability distribution over possible canopy heights, given the input data, and estimating the mean $\hat{\mu }$ and variance ${\hat{\sigma }}^{2}$ of that distribution.

1 $${{{{\mathcal{L}}}}}_\mathrm{NLL}=\frac{1}{N}\mathop{\sum }\limits_{i=1}^{N}\frac{{\left(\,{\hat{\mu} }({x}_{i})-{y}_{i}\right)}^{2}}{2{\hat{\sigma }}^{2}({x}_{i})}+\frac{1}{2}\log {\hat{\sigma }}^{2}({x}_{i}).$$

To account for the model uncertainty, which in high-capacity neural network models can be interpreted as the model’s lack of knowledge about patterns not adequately represented in the training data, we train an ensemble^29^ of five CNNs from scratch, that is, each time starting the training from a different randomly initialized set of model weights (learnable parameters). At inference time, we process images from *T* different acquisition dates (here *T* = 10) for every location to obtain full coverage and to exploit redundancy in the case of repeated cloud-free observations of a pixel. Each image is processed with one CNN picked randomly from the ensemble. This procedure incurs no additional computational cost compared to processing all images with the same CNN. It can be interpreted as a natural variant of test-time augmentation, which has been demonstrated to improve the calibration of uncertainty estimates from deep ensembles in the domain of computer vision^37^. Finally, the per-image estimates are merged into a final map by averaging with inverse-variance weighting (equation (3)). If the variance estimates of all ensemble members are well calibrated, this results in the lowest expected error^38^. Thus the variance of the final per-pixel estimate is computed with the weighted version of the law of total variance (equation (4))^35^. For readability we omit the pixel index *i*.

2 $${\hat{p}}_{t}=\frac{1/{\hat{\sigma }}_{t}^{2}}{\mathop{\sum }\nolimits_{{j = 1}}^{\quad \, \, {T}}1/{{\hat{\sigma }}_{j}}^{2}},$$

3 $${\hat{y}}=\mathop{\sum }\limits_{t=1}^{T}{\hat{p}}_{t}{\hat{\mu }}_{t},$$

4 $${{{\rm{Var}}}}(\,{\hat{y}})=\mathop{\sum }\limits_{t=1}^{T}{\hat{p}}_{t}{\hat{\mu }}_{t}^{2}-{\left(\mathop{\sum }\limits_{t = 1}^{T}{\hat{p}}_{t}{\hat{\mu }}_{t}\right)}^{2}+\mathop{\sum }\limits_{t=1}^{T}{\hat{p}}_{t}{\hat{\sigma }}_{t}^{2},$$

### Correction for imbalanced height distribution

We find that the underestimation bias on tall canopies is partially due to the imbalanced distribution of reference labels (and canopy heights overall), where large height values occur rarely. To mitigate it, we fine tune the converged model with a cost-sensitive version of the loss function. A softened version of inverse sample-frequency weighting is used to re-weight the influence of individual samples on the loss (equation (5)). To establish the frequency distribution of the continuous canopy height values in the training, we bin them into 1-m height intervals and in each of the resulting *K* bins count the number of samples *N*_*k*_. Empirically, for our task, the moderated reweighting with the square root of the inverse frequency works better (leaving all other hyper-parameters unchanged). Moreover, we do not fine tune all model parameters but only the final regression layer that computes mean height (Fig. 1a). We observe that the uncertainty calibration is preserved when fine tuning only the regression weights for the mean (‘S2 + geo balanced: mean’ in Extended Data Fig. 5a), whereas fine tuning also the regression of the variance decalibrates the uncertainty estimation (‘S2 + geo balanced: mean&var’). The fine tuning is run for 750,000 iterations per network.

5 $${q}_{i}=\frac{\sqrt{1/{N}_{k,i\in k}}}{\mathop{\sum }\nolimits_{j = 1}^{K}\sqrt{1/{N}_{j}}}$$

### Evaluation metrics

Several metrics are employed to measure prediction performance: the RMSE (equation (6)) of the predicted heights, their MAE (equation (7)) and their mean error (ME, equation (8)). The latter quantifies systematic height bias, where a negative ME indicates underestimation, that is, predictions that are systematically lower than the reference values.

6 $${{{\rm{RMSE}}}}=\sqrt{\frac{1}{N}\mathop{\sum }\limits_{i=1}^{N}{\left({\hat{y}}_{i}-{y}_{i}\right)}^{2}}$$

7 $${{{\rm{MAE}}}}=\frac{1}{N}\mathop{\sum }\limits_{i=1}^{N}|\, {\hat{y}}_{i}-{y}_{i}|$$

8 $${{{\rm{ME}}}}=\frac{1}{N}\mathop{\sum }\limits_{i=1}^{N}\left({\hat{y}}_{i}-{y}_{i}\right)$$

We also report balanced versions of these metrics, where the respective error is computed separately in each 5-m height interval and then averaged across all intervals. They are abbreviated as aRMSE, aMAE and aME (Fig. 1b).

The bias analyses with the independent airborne LiDAR data include the normalized mean error (NME, equation (7)) in percentage, where the mean error is divided by the average of the reference values $\bar{y}$:

9 $${{{\rm{NME}}}}=\frac{100}{\bar{y}N}\mathop{\sum }\limits_{i=1}^{N}\left({\hat{y}}_{i}-{y}_{i}\right)$$

For the estimated predictive uncertainties, there are, by definition, no reference values. A common scheme to evaluate their calibration is to produce calibration plots^34,57^ that show how well the uncertainties correlate with the empirical error. As this correlation holds only in expectation, both the uncertainties and the empirical errors at the test samples must be binned into *K* equally sized intervals. In each bin *B*_*k*_, the average of the predicted uncertainties is then compared against the actual average deviation between the predicted height and the reference data. On the basis of the calibration plots, it is further possible to derive a scalar error metric for the uncertainty calibration, the uncertainty calibration error (UCE) (equation (10))^57^. Again, we additionally report a balanced version, the average uncertainty calibration error (AUCE) (equation (11)), where each bin has the same weight independent of the number *N*_*k*_ of samples in it.

10 $${{{\rm{UCE}}}}=\mathop{\sum }\limits_{k=1}^{K}\frac{{N}_{k}}{N}| \mathrm{err}({B}_{k})-\mathrm{uncert}({B}_{k})|$$

11 $${{{\rm{AUCE}}}}=\frac{1}{K}\mathop{\sum }\limits_{k=1}^{K}| \mathrm{err}({B}_{k})-\mathrm{uncert}({B}_{k})|$$

In our case err(*B*_*k*_) represents the RMSE of the samples falling into bin *B*_*k*_, and the bin uncertainty uncert(*B*_*k*_) is defined as the root mean variance (RMV):

12 $${{{\rm{RMV}}}}=\sqrt{\frac{1}{{N}_{k}}\mathop{\sum}\limits_{i\in {B}_{k}}{\hat{u}}_{i}},$$

with ${\hat{u}}_{i}={\hat{\sigma}}_{i}^{2}$ when evaluating the calibration of a single CNN and ${\hat{u}}_{i}={{{\rm{Var}}}}(\,{\hat{y}}_{i})$ when evaluating the calibration of the ensemble. We refer to the RMV as the predictive standard deviation in our calibration plots (Extended Data Fig. 5a,c).

### Global map computation

Sentinel-2 imagery is organized in 100 km × 100 km tiles; a total of 18,011 tiles cover the entire landmass of the Earth, excluding Antarctica. However, depending on the ground tracks of the satellites, some tiles are covered by multiple orbits, whereas, in general, no more than two orbits are needed to get full coverage. To optimize computational overhead, we select the relevant orbits per tile by using those with the smallest number of empty pixels, according to the metadata. For every tile and relevant orbit, the ten images with least cloud cover between May and September 2020 are selected for processing.

While it only takes ≈ 2 minutes to process a single image tile with the CNN on a GeForce RTX 2080 Ti GPU, downloading the image from the Amazon Web Service S3 bucket takes about 1 minute, and loading the data into memory takes about 4 minutes. To process a full tile with all ten images per orbit takes between 1 and 2.5 hours, depending on the number of relevant orbits (one or two).

We apply only minimal post-processing and mask out built-up areas, snow, ice and permanent water bodies according to the ESA WorldCover classification^58^, setting their canopy height to ‘no data’ (value: 255). The canopy height product is released in the form of 3° × 3° tiles in geographic longitude/latitude, following the format of the recent ESA WorldCover product. This choice shall simplify the integration of our map into existing workflows and the intersection of the two products. Note that the statistics in the present paper were not computed from those tiles but in Gall–Peters orthographic equal-area projection with 10-m GSD for exact correspondence between pixel counts and surface areas.

### Energy and carbon emissions footprint

The presented map has been computed on a GPU cluster located in Switzerland. Carbon accounting for electricity is a complex endeavour, due to differences in how electricity is produced and distributed. To put the power consumption needed to produce global maps with our method into context, we estimate carbon emissions for two scenarios, where the computation is run on Amazon Web Services (AWS) in two different locations: European Union (Stockholm) and United States East (Ohio). With ≈ 250 W to power one of our GPUs, we get a total energy consumption of 250 W × 27,000 h = 6,750 kWh for the global map. The conversion to emissions highly depends on the carbon efficiency of the local power grid. For European Union (Stockholm), we obtain an estimated 338 kg CO_2_-equivalent, whereas for United States East (Ohio), we obtain 3,848 kg CO_2_-equivalent, a difference by a factor >10. Whereas the former is comparable to driving an average car from Los Angeles to San Francisco and back (1,360 km), the latter corresponds to a round trip from Seattle (United States) to San José, Costa Rica (15,500 km). These estimates were conducted using the Machine Learning Impact calculator (ref. ^59^). For the carbon footprint of the current map (not produced with AWS), we estimate ≈ 729 kg CO_2_-equivalent, using an average of 108 g CO_2_-equivalent kWh^−1^ for Switzerland, as reported for the year 2017^60^.

### Reporting summary

Further information on research design is available in the Nature Portfolio Reporting Summary linked to this article.

### Supplementary information

Supplementary Information Supplementary Figs. 1–5.

Reporting Summary

Peer Review File

## Figures and Tables

**Extended Data Table 1:**
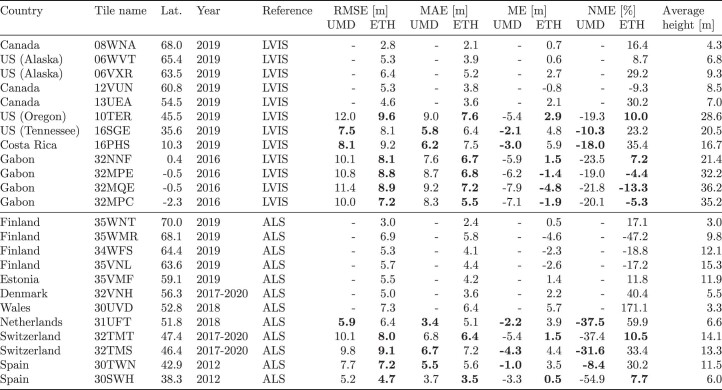
Tile-level evaluation with independent airborne LIDAR data

LVIS airborne LIDAR (Canada, US, Costa Rica, and Gabon) and GEDI-like canopy top height derived from small-footprint airborne laser scanning (ALS) campaigns in Europe (Finland, Estonia, Denmark, Wales, Netherlands, Switzerland, and Spain). Error metrics are given per Sentinel-2 tile for a total of 24 tiles, 12 tiles for LVIS (top rows) and 12 tiles for ALS (bottom rows). The best results are highlighted in bold.

**Extended Data Table 2:**
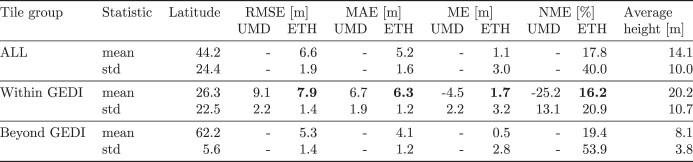
Averaged evaluation with independent airborne LIDAR data

Comparison with independent airborne LIDAR data (LVIS and ALS from Extended Data Table 1) averaged over all 24 tiles (top), tiles within the GEDI coverage (center), and tiles beyond the GEDI coverage (bottom).

## Data Availability

A summary of all links to data, browser application and code can be found on the project page at langnico.github.io/globalcanopyheight. Global map: the global canopy height map for 2020 is available for download at 10.3929/ethz-b-000609802. Individual tiles can be downloaded at langnico.github.io/globalcanopyheight/assets/tile_index.html. The map is also available on the Google Earth Engine (GEE assets on project page). The global map can be explored interactively in this browser application: nlang.users.earthengine.app/view/global-canopy-height-2020. GEDI footprint data: sparse footprint-level RH98 estimates used as reference data for developing the presented model are available on Zenodo at 10.5281/zenodo.7737946 (ref. ^53^), which is the filtered version of the full orbit predictions for 2019, 10.5281/zenodo.5704852 (ref. ^61^), and 2020, 10.5281/zenodo.7737869 (ref. ^62^). Training and validation datasets: the global training and validation dataset with image patches and rastered reference data is available at 10.3929/ethz-b-000609845. The rastered airborne LiDAR canopy height models from LVIS and ALS used for validation are available at 10.5281/zenodo.7885699. ESA WorldCover: a derived version of the original ESA WorldCover 10-m 2020 v100^58^ re-projected to the Sentinel-2 UTM Tiling Grid for the global land surface is available at 10.5281/zenodo.7888150 (ref. ^63^).

## Code availability

The source code and the trained models are available via Github, github.com/langnico/global-canopy-height-model.
