# Publisher Correction: A high-resolution canopy height model of the Earth

**DOI:** 10.1038/s41559-024-02371-2

**Published:** 2024-02-19

**Authors:** Nico Lang, Walter Jetz, Konrad Schindler, Jan Dirk Wegner

**Affiliations:** 1 EcoVision Lab, Photogrammetry and Remote Sensing, ETH Zürich, Zürich, Switzerland; 2 Department of Computer Science, University of Copenhagen, Copenhagen, Denmark; 3 Department of Ecology and Evolutionary Biology, Yale University, New Haven, CT USA; 4 Institute for Computational Science, University of Zurich, Zürich, Switzerland

**Keywords:** Environmental impact, Ecological modelling, Biogeography, Forest ecology

Correction to: *Nature Ecology & Evolution* 10.1038/s41559-023-02206-6, published online 28 September 2023.

In the version of the article initially published, the top label of the scale bar in Fig. 2a read “>15” but has now been amended to “>50” in the HTML and PDF versions of the article.

