# Supplementary material for: A high-resolution canopy height model of the Earth
Source: Nat Ecol Evol. 2023 Sep 28;7(11):1778–89. doi: 10.1038/s41559-023-02206-6 (PMC10627820; doi:10.1038/s41559-023-02206-6)
Supplement: Supplementary file 1 — Supplementary Figs. 1–5. [file 41559_2023_2206_MOESM1_ESM.pdf]

nature ecology & evolution

Article

https://doi.org/10.1038/s41559-023-02206-6

A high-resolution canopy height model of the Earth

In the format provided by the authors and unedited

Supplementary Information Figures

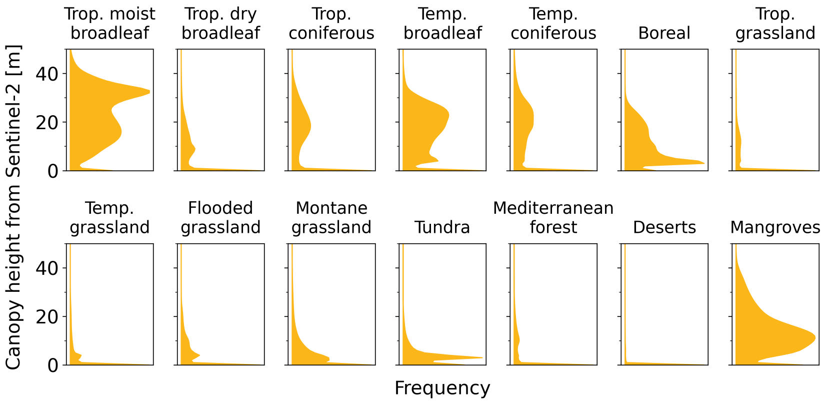

(a)

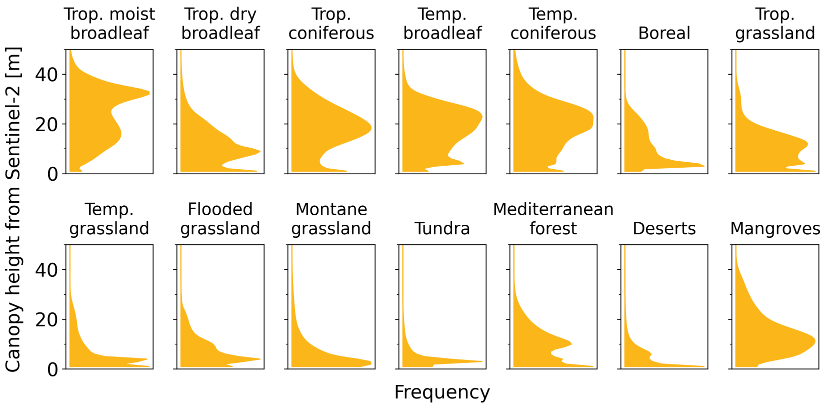

(b)

Supplementary Figure 1: Biome-level frequency distribution of canopy heights according to 14 terrestrial ecosystems defined by The Nature Conservancy. Urban areas and croplands (based on ESA World Cover [40]) have been excluded. a) Same as Fig. 3b (i.e. including the zero height bin). b) Same as Fig. 3b, but only using canopy top heights >1 m (i.e. excluding the zero height bin).

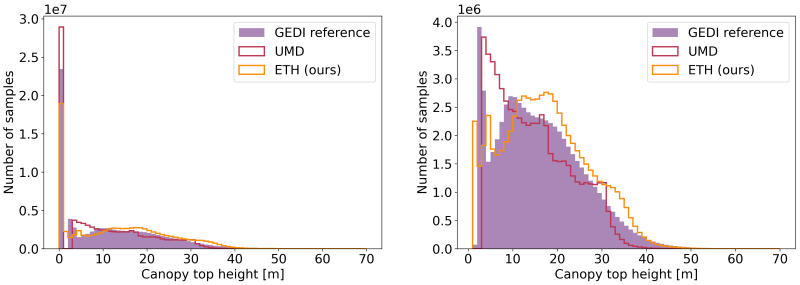

(a) (b)

Supplementary Figure 2: Distribution of canopy top height at GEDI validation locations comparing GEDI reference data against UMD estimates and our estimates. a) Canopy top height >0 m b) Canopy top height >1 m (i.e. excluding the zero height bin). GEDI shots are not a uniform sample of the land surface. Thus, the distributions of sparse GEDI data is not comparable with the distribution of our estimated dense data (Fig. 3).

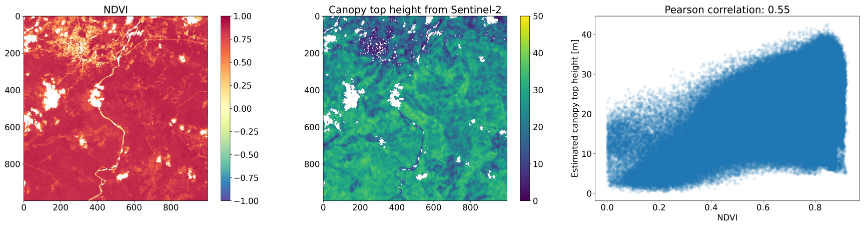

Supplementary Figure 3: Correlation between the normalized difference vegetation index (NDVI) and estimated canopy top height from Sentinel-2. An example area of 10 km×10 km from the LVIS region in Costa Rica (tile name 16PHS). Although there is a positive correlation between NDVI and the estimated canopy height, the learned relationship between the optical image features (textural and spectral) and the canopy top height are more complex and cannot easily be described by a standard spectral index such as the NDVI.

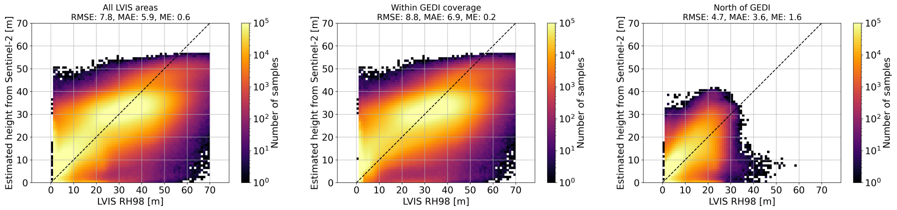

(a) (b) (c)

Supplementary Figure 4: Evaluation w.r.t. canopy top height (RH98) derived from independent LVIS airborne LIDAR data [32]. a-c) Confusion plots showing the relationship between LVIS reference data and predictions from Sentinel-2 for a) All available LVIS areas, b) Only regions within the GEDI range, and c) Only regions north of GEDI.

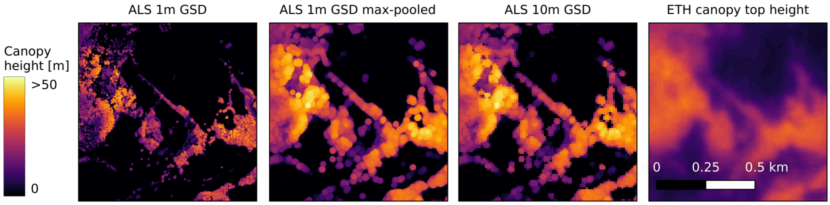

Supplementary Figure 5: Illustration of the ALS data processing. From left to right: Canopy height model at 1 m ground sampling distance (GSD) derived from small-footprint airborne laser scanning (ALS) campaigns. Dense "GEDI-like" canopy top height max-pooled within the 25 m footprint at every 1 m pixel. Final reference canopy height model bilinearly resampled to the Sentinel-2 10 m GSD. Our canopy top height estimates (10 m GSD) from Sentinel-2 images.
