# Supplementary material for: A high-resolution canopy height model of the Earth
Source: Nat Ecol Evol. 2023 Sep 28;7(11):1778–89. doi: 10.1038/s41559-023-02206-6 (PMC10627820; doi:10.1038/s41559-023-02206-6)
Supplement: Supplementary file 2 — Reporting Summary [file 41559_2023_2206_MOESM2_ESM.pdf]

nature portfolio

Corresponding author(s): Nico Lang, Jan Dirk Wegner

Last updated by author(s): May 11, 2022

Reporting Summary

Nature Portfolio wishes to improve the reproducibility of the work that we publish. This form provides structure for consistency and transparency in reporting. For further information on Nature Portfolio policies, see our Editorial Policies and the Editorial Policy Checklist.

Statistics

For all statistical analyses, confirm that the following items are present in the figure legend, table legend, main text, or Methods section.

n/a	Confirmed	
☐	☒	The exact sample size (*n*) for each experimental group/condition, given as a discrete number and unit of measurement
☒	☐	A statement on whether measurements were taken from distinct samples or whether the same sample was measured repeatedly
☒	☐	The statistical test(s) used AND whether they are one- or two-sided<br>*Only common tests should be described solely by name; describe more complex techniques in the Methods section.*
☐	☒	A description of all covariates tested
☒	☐	A description of any assumptions or corrections, such as tests of normality and adjustment for multiple comparisons
☐	☒	A full description of the statistical parameters including central tendency (e.g. means) or other basic estimates (e.g. regression coefficient) AND variation (e.g. standard deviation) or associated estimates of uncertainty (e.g. confidence intervals)
☒	☐	For null hypothesis testing, the test statistic (e.g. *F*, *t*, *r*) with confidence intervals, effect sizes, degrees of freedom and *P* value noted<br>*Give P values as exact values whenever suitable.*
☒	☐	For Bayesian analysis, information on the choice of priors and Markov chain Monte Carlo settings
☒	☐	For hierarchical and complex designs, identification of the appropriate level for tests and full reporting of outcomes
☒	☐	Estimates of effect sizes (e.g. Cohen's *d*, Pearson's *r*), indicating how they were calculated

Our web collection on statistics for biologists contains articles on many of the points above.

Software and code

Policy information about availability of computer code

Data collection	The data were provided by Copernicus/ESA and NASA's GEDI mission. Sentinel-2 images were accessed using the sentinelsat and sentinelhub API from Scihub and AWS S3. GEDI-derived data were accessed from zenodo.
Data analysis	Only free and open source software was used for data analysis: python 3.7, pytorch '1.8.0+cu101', QGIS 3.20, GDAL 3.2.0

For manuscripts utilizing custom algorithms or software that are central to the research but not yet described in published literature, software must be made available to editors and reviewers. We strongly encourage code deposition in a community repository (e.g. GitHub). See the Nature Portfolio guidelines for submitting code & software for further information.

Data

Policy information about availability of data

All manuscripts must include a data availability statement. This statement should provide the following information, where applicable:

- Accession codes, unique identifiers, or web links for publicly available datasets
- A description of any restrictions on data availability
- For clinical datasets or third party data, please ensure that the statement adheres to our policy

The global canopy height map for 2020 is accessible for download and available in the Google Earth Engine. All links, source code, and the trained models used to generate the map will be released via the project page: https://langnico.github.io/globalcanopyheight/. The global map can be explored interactively in this browser application: https://nlang.users.earthengine.app/view/global-canopy-height-2020

Field-specific reporting

Please select the one below that is the best fit for your research. If you are not sure, read the appropriate sections before making your selection.

☐ Life sciences ☐ Behavioural & social sciences ☒ Ecological, evolutionary & environmental sciences

For a reference copy of the document with all sections, see nature.com/documents/nr-reporting-summary-flat.pdf

Ecological, evolutionary & environmental sciences study design

All studies must disclose on these points even when the disclosure is negative.

Study description	A deep learning model was trained on ~600,000 GEDI footprints paired with the corresponding Sentinel-2 image patches. The global canopy height map is based on 160 TB of Sentinel-2 image data from the year 2020.
Research sample	The study is a wall-to-wall assessment of the global landmass at 10-meter ground sampling distance.
Sampling strategy	No sampling was necessary as the entire landmass is part of the study. For the evaluation of the model performance, the sampling is randomized at the Sentinel-2 tile level (100 km x 100 km regions). The sampling with the tiles is given by the GEDI sampling pattern along the ground tracks of the International Space Station.
Data collection	Sentinel-2 images were downloaded from the AWS S3 bucket. GEDI-derived data were accessed from zenodo. LVIS airborne LIDAR data is downloaded from the National Snow & Ice Data Center (NSIDC).
Timing and spatial scale	The global canopy height map is based on images from the year 2020. For every location, we processed the 10 image tiles with the least cloud coverage between May and September.
Data exclusions	We have used the ESA World Cover Map to mask urban and water areas. In the biome-level distribution analyses, we have exlcuded crop lands based on the ESA World Cover Map to characterize the height distribution of natural ecosystems.
Reproducibility	The final map is the result of an ensemble of five models deployed on 10 repeated image observations. As the optimization of deep neural networks is based on stochastic algorithms, slight variations in the output are to be expected.
Randomization	NA. To evaluate the model globally, we split the collected dataset at the level of Sentinel-2 tiles. I.e., of the 100 km×100 km regions defined by the Sentinel-2 tiling 20% are held out for validation, the remaining 80% are used to train the model.
Blinding	NA. We additionally report an evaluation of our final model against independent reference data from NASA's LVIS airborne LIDAR campaigns.

Did the study involve field work? ☐ Yes ☒ No

Reporting for specific materials, systems and methods

We require information from authors about some types of materials, experimental systems and methods used in many studies. Here, indicate whether each material, system or method listed is relevant to your study. If you are not sure if a list item applies to your research, read the appropriate section before selecting a response.

Materials & experimental systems

n/a	Involved in the study	
☒	☐	Antibodies
☒	☐	Eukaryotic cell lines
☒	☐	Palaeontology and archaeology
☒	☐	Animals and other organisms
☒	☐	Human research participants
☒	☐	Clinical data
☒	☐	Dual use research of concern

Methods

n/a	Involved in the study	
☒	☐	ChIP-seq
☒	☐	Flow cytometry
☒	☐	MRI-based neuroimaging
